# *In silico* directed mutagenesis identifies the CD81/claudin-1 hepatitis C virus receptor interface

**DOI:** 10.1111/cmi.12008

**Published:** 2012-09-25

**Authors:** Christopher Davis, Helen J Harris, Ke Hu, Heidi E Drummer, Jane A McKeating, Jonathan G L Mullins, Peter Balfe

**Affiliations:** 1 School of Immunity and Infection, Institute of Biomedical Research, College of Medical and Dental Sciences, University of BirminghamBirmingham, B15 2TT, UK; 2 Viral Fusion Laboratory, Burnet InstituteMelbourne, Vic., 3004, Australia; 3 Department of Microbiology, Monash UniversityClayton, Vic., 3800, Australia; 4 Department of Microbiology and Immunology, Melbourne UniversityParkville, Vic., 3010, Australia; 5 NIHR Liver Biomedical Research Unit, University of BirminghamBirmingham, B15 2TT, UK; 6 Genome and Structural Bioinformatics, Institute of Life Science, College of Medicine, Swansea UniversitySwansea, SA2 8PP, Wales, UK

## Abstract

Hepatitis C virus (HCV) entry is dependent on host cell molecules tetraspanin CD81, scavenger receptor BI and tight junction proteins claudin-1 and occludin. We previously reported a role for CD81/claudin-1 receptor complexes in HCV entry; however, the molecular mechanism(s) driving association between the receptors is unknown. We explored the molecular interface between CD81 and claudin-1 using a combination of bioinformatic sequence-based modelling, site-directed mutagenesis and Fluorescent Resonance Energy Transfer (FRET) imaging methodologies. Structural modelling predicts the first extracellular loop of claudin-1 to have a flexible beta conformation and identifies a motif between amino acids 62–66 that interacts with CD81 residues T149, E152 and T153. FRET studies confirm a role for these CD81 residues in claudin-1 association and HCV infection. Importantly, mutation of these CD81 residues has minimal impact on protein conformation or HCVglycoprotein binding, highlighting a new functional domain of CD81 that is essential for virus entry.

## Introduction

Many viruses initiate infection through a multistep process involving multiple host cell receptor proteins (for review, see Grove and Marsh, 2011). Although the molecular interaction of viruses with individual receptor proteins has been elucidated in great detail, the more dynamic process of multi-protein engagement has proven a greater challenge. The sequential engagement of receptors provides a co-ordinated process to regulate virus internalization and offers new targets for anti-viral intervention.

Hepatitis C virus (HCV) interacts with tetraspanin CD81 and scavenger receptor BI (SR-BI) (Pileri *et al*., 1998; Scarselli *et al*., 2002) and permissive cells express tight junction proteins claudin-1 and occludin, epidermal growth factor receptor and Niemann-Pick C1-like 1 protein (Burlone and Budkowska, 2009; Ploss *et al*., 2009; Dorner *et al*., 2011; Lupberger *et al*., 2011; Meredith *et al*., 2012; Sainz *et al*., 2012), highlighting the multi-step nature of the internalization process. At the present time, the role these individual entry factors play and how they co-ordinate HCV entry is unknown (Evans *et al*., 2007; Cukierman *et al*., 2009; Krieger *et al*., 2010).

CD81 was the first cellular protein identified to bind HCV and is a member of the tetraspanin family of proteins that are widely expressed and involved in multiple biological functions, including cell proliferation and cell–cell adhesion (Hemler, 2005). The diverse role of tetraspanins in the infectivity of various pathogens, including the malarial plasmodium parasite, HIV-1, Influenza and human papilloma virus, and in cancer prompted a search for ligands, antibodies or small molecules that selectively inhibit tetraspanin function (Spoden *et al*., 2008; Hassuna *et al*., 2009; Wang *et al*., 2011). Although several antibodies targeting the CD81 second extracellular loop (ECL2) have been reported to inhibit HCV infection *in vitro* and *in vivo* (Meuleman *et al*., 2008; Farquhar *et al*., 2012), they have also been shown to promote immune cell proliferation and to activate multiple cellular processes, limiting their therapeutic potential (Levy and Shoham, 2005; Coffey *et al*., 2009).

In contrast, there is limited evidence for tight junction protein association with HCV, suggesting an indirect role for claudin-1 and occludin in virus internalization (Meredith *et al*., 2012). We (Harris *et al*., 2008; 2010) and others (Kovalenko *et al*., 2007; Cukierman *et al*., 2009) have shown a direct interaction of claudins with tetraspanins, supporting a model where CD81–claudin-1 receptor complexes define HCV internalization. The claudin superfamily of four transmembrane proteins play an essential role in the formation and maintenance of tight junctions in epithelial and endothelial cells. However, claudin-1 has been reported to associate with CD81 at the basolateral surface of polarized hepatoma cells, suggesting a role for non-junctional pools of claudin-1 in HCV entry (Harris *et al*., 2010). Mutagenesis of the first claudin-1 extracellular loop (ECL1) ablated both its association with CD81 and HCV entry (Evans *et al*., 2007; Cukierman *et al*., 2009; Harris *et al*., 2010), illustrating the essential role of the receptor complex in HCV infection. Furthermore, antibodies specific for claudin-1 ECL1 inhibit HCV infection by reducing claudin-1 association with CD81 (Fofana *et al*., 2010; Krieger *et al*., 2010), highlighting the therapeutic value of targeting the receptor complex to limit infection.

We investigated the molecular interface between CD81 and claudin-1 using a combination of bioinformatic sequence-based modelling, site-directed mutagenesis and Fluorescent Resonance Energy Transfer (FRET). Although the crystal structure of human CD81 ECL2 is known (PDB 1G8Q) (Kitadokoro *et al*., 2001; Drummer *et al*., 2002; 2005), no X-ray or NMR structural data exists for any member of the claudin family. We generated a homology model for claudin-1 ECL1 that predicts amino acid regions 33–35 and 62–66 to associate with CD81 ECL2 residues T149, E152 and T153. We used this model to guide the genesis and *in vitro* screening of a panel of claudin-1 and CD81 mutants. We demonstrate an essential role for CD81 residues T149, E152 and T153 to associate with claudin-1. Importantly, these mutations had no impact on protein conformation or HCV E2 binding and ablate HCV entry, highlighting the novel function of these CD81 residues. These observations increase our understanding of CD81/claudin-1 association and provide a platform for the computational design of peptides and small molecules to disrupt receptor complexes and inhibit viral infection.

## Results

### Modelling claudin-1 ECL1 and its interaction with CD81

**Fig. 1:**
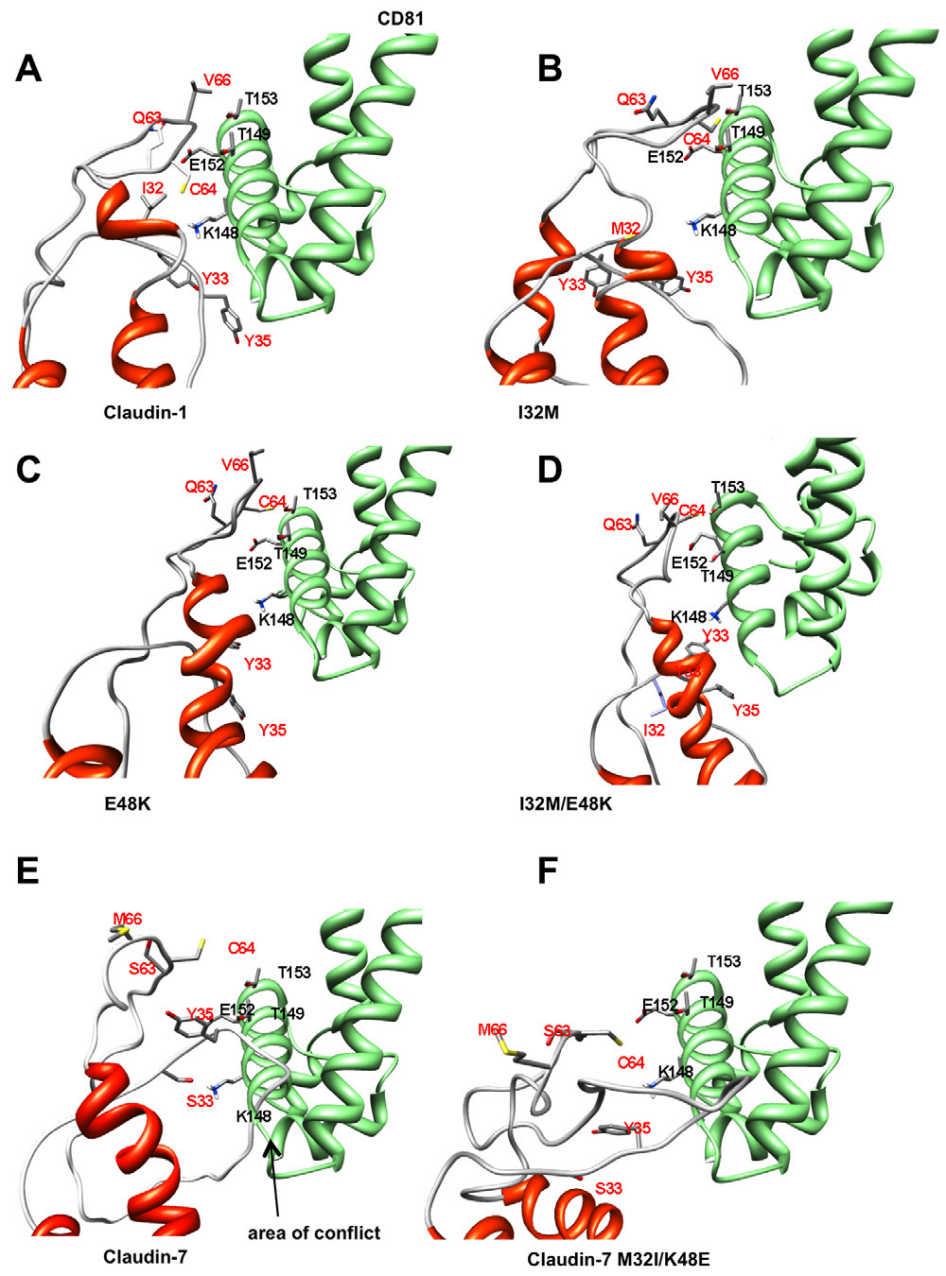
Structural modelling of claudin-CD81 association. Ribbon models of the ECL1 domain of native claudin-1 (A) or I32M (B), E48K (C) and I32M/E48K (D) mutants with CD81 ECL2 (PDB: 1G8Q). Claudin-1 is depicted according to its predicted secondary structure (alpha helices in red, beta turn in white) with CD81 in green, key interacting residues are labelled. The interacting regions include claudin-1 residues 33–35 and 63–66, and CD81 residues K148, T149, E152 and T153. I32M and E48K claudin-1 mutations alter key inter-residue distances and interactions with CD81. Model of the ECL1 domain of native claudin-7 (E) or M32I/K48E mutant (F) with the CD81 ECL. Mutagenesis of claudin-7 relocates the loop around C64 to a position similar to that of claudin-1 and withdraws a prominent loop away from the region of claudin-CD81 interaction. Images were produced using the Chimera program (University of San Francisco).

We previously reported that recombinant claudin-1 ECL1 bound dimeric CD81 ECL2 (Harris *et al*., 2010), demonstrating an association between the extracellular loops of these two proteins. A blast search of claudin-1 against all sequences with determined structures in the PDB database revealed limited homology; however, a 30% identity was found between claudin-1 residues 4–57 and residues 21–74 of Cj0915, a hexameric hotdog fold thioesterase of *Campylobacter jejuni* (PDB:3D6L), suggesting that ECL1 adopts a flexible beta conformation. Lacking a full-length homologue, we widened our search to include theoretical structures. This search identified the only published model of a tetraspanin, CD81 (PDB: 2AVZ; Seigneuret, 2006) as the most suitable whole length homologue, with a sequence similarity of 45% along the 211 amino acid length of the protein. We used this CD81 template, including the crystal structure co-ordinates of ECL2 (Kitadokoro *et al*., 2001), to predict a structure for claudin-1 ECL1 tethered at either end to transmembrane regions appropriately packed within a four transmembrane region bundle (Fig. 1A).

Our model predicts claudin-1 ECL1 to consist mostly of beta turns with few structural constraints and identifies two regions that interface with CD81 ECL2 (Fig. 1A). The first region (amino acids 33–35) orientates the loops to form a stable complex, whereas the second (amino acids 63–66) interacts directly with K148, T149, E152 and T153 of CD81 (Fig. 1A). The surface area of the CD81/claudin-1 ECL interface was calculated to be 1005 Å^2^, representing 7% of the accessible surface area of each protein.

To evaluate our CD81/claudin-1 model we studied the effect of HCV receptor inactivating claudin-1 mutations I32M and E48K (Evans *et al*., 2007), previously reported to ablate claudin-1 association with CD81 (Harris *et al*., 2010). Molecular models of I32M (Fig. 1B) and E48K (Fig. 1C) mutants predicted substantial changes in the structure and orientation of ECL1, with the introduction of a short α helix and increased distances between CD81 interacting residues. Most markedly, for the claudin-1 I32M mutant, the Q63 – CD81 E152 distance increased to over 10 Å, leading to a dramatic loss of surface area for protein interaction. Similarly, mutating claudin-1 E48K induced a loss of three of the four key residue pairings, suggesting a profound impact on loop orientation and diminished association with CD81. The double I32M/E48K mutant induced significant changes in ECL1 orientation and interaction with CD81 contact residues (Fig. 1D). We previously reported that receptor inactive claudin-7 does not interact with CD81, however, mutation of residues M32I and K48E conferred viral receptor activity and CD81 association (Harris *et al*., 2010). *In silico* modelling of claudin-7 predicted no association with CD81 (Fig. 1E). However, the double mutant adopted a structure closer to that of claudin-1 in which claudin-7 C64 was positioned to interact with CD81 E152 (Fig. 1F).

**Fig. 2:**
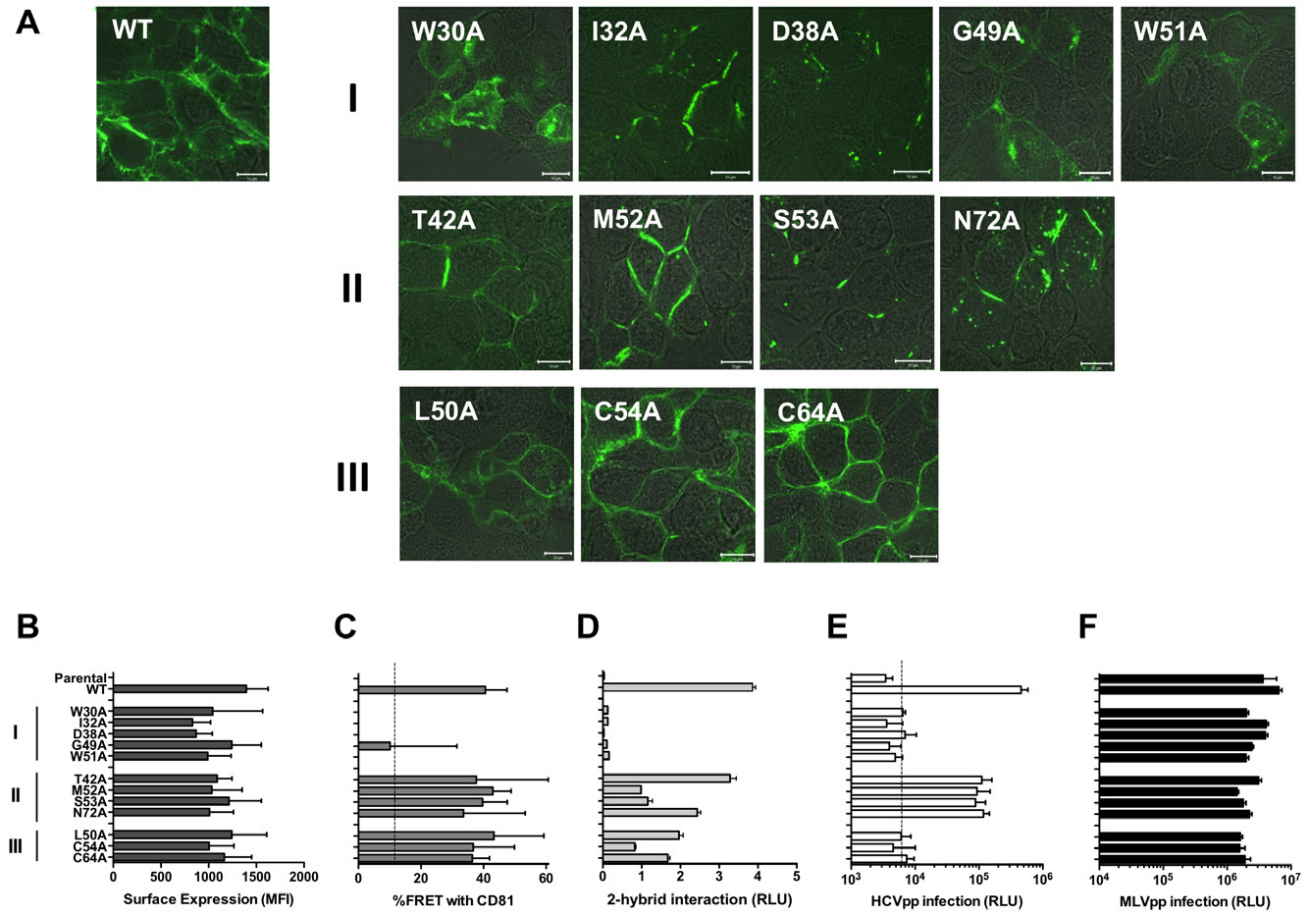
Effect of claudin-1 mutations on protein localization, CD81 association and HCV infection. A. 293-T cells were transfected to express wild-type (WT) AcGFP–claudin-1 and mutants and their localization assessed by confocal microscopy. B. Cell surface expression of AcGFP–claudin mutants was determined using the Zeiss profiling function to trace cell boundaries, the data shown is the average MFI of 10 cell profiles, the data are representative of three transfection experiments. C. %FRET between AcGFP–claudin-1 mutants and DsRED-CD81 in transfected 293-T cells. None of the mutants in group I (W30A, I32A, D38A, G49A and W51A) showed any significant interaction with CD81. The mean %FRET values are derived from triplicate estimates within a single experiment and are representative of two further experiments. The mean plus 2 SD of non-specific FRET values observed in parental non-transfected is indicated by the dashed line and represents the threshold for the assay. The diminution in %FRET signal for the members of group I relative to the other two groups was significant (Kruskal Wallis test, *P* < 0.01). D. Two-hybrid screening of interactions between mutant claudin-1 and WT CD81. None of the claudin-1 mutants in group I showed evidence for association *in vitro*. E and F. Infectivity of HCVpp (E) and MLVpp (F) in parental 293-T cells and those transfected to express AcGFP–claudin-1 WT and mutants. 293-T cells expressed comparable levels of WT and claudin-1 mutants. Data are expressed as specific infectivity where the value of an envelope deficient pseudoparticle is subtracted from both HCVpp and MLVpp relative light unit (RLU) signals. The dashed line represents the mean plus 2 SD of HCVpp infection of claudin-1-negative parental 293-T cells; levels of infection below this threshold are considered negative. The data presented are from a single experiment and are representative of two independent experiments.

To further study our CD81/claudin-1 model we studied a panel of claudin-1 mutants, previously reported to disrupt HCV receptor activity (Cukierman *et al*., 2009), for their effect(s) on claudin-1 ECL1 structure and association with CD81 (Harris *et al*., 2010). *In silico* screening classified the claudin-1 mutants into three groups: **group I** mutants W30A, I32A, I32M, D38A, E48K, G49A and W51A, showed a withdrawal or disruption of interfacing regions and no association with CD81; **group II** mutants T42A, M52A, S53A and N72A, showed minimal structural alteration(s) and a continued association with CD81; and **group III** mutants L50A, C54A and C64A, showed an altered structure and association with different CD81 residues L162, I182, N184 and F186 (Table 1). To assess claudin-1 mutant association with CD81, we expressed N-terminal AcGFP–claudin fusion proteins to quantify expression and interaction with DsRED-CD81. All tagged claudin-1 mutants localized at the plasma membrane (Fig. 2A) and exhibited similar levels of surface expression (Fig. 2B). FRET studies confirmed our *in silico* predictions of the effect(s) of claudin-1 mutations on CD81 association. Group I mutants showed no significant FRET with CD81, whereas group II and III mutants showed similar FRET values to wild-type (WT) claudin-1 (Fig. 2C). All of the mutants were co-transfected as fusion constructs in a two-hybrid screen with a WT CD81 ‘trap’, these assays showed that all of the claudin-1 molecules in groups II and III associated with CD81 whereas members of group I failed to interact (Fig. 2D). The mutants supported varying levels of HCVpp infection (Fig. 2E). Importantly, all group I mutants lacked viral receptor activity whereas group II mutants supported HCVpp entry at comparable levels to WT claudin-1 (Fig. 2E). In contrast, HCVpp failed to infect 293-T cells expressing group III claudin-1 mutants, demonstrating that the altered claudin-1-CD81 interaction does not support HCV entry (Table 1). In summary, these results validate our model and demonstrate the value of a structural modelling approach to predict the functional impact of claudin-1 mutations on HCVpp infection.

**Table 1:** Predicted effect(s) of claudin-1 mutations on CD81:claudin-1 association

	HCV receptor activity	ECL1 orientation^a^	Disruption of Q63-V66 and withdrawal from E152	Orientation of interacting residues disrupted	Predicted to interact with CD81	Mutant grouping
Claudin-1 mutation						
WT	+	WT	No	No	Yes	–
W30A	−	Projecting	Yes	Yes	No	I
I32A	−	Projecting	Yes	Yes	No	I
I32M	−	Withdrawn	Yes	Yes	No	I
D38A	−	Withdrawn	Yes	Yes	No	I
E48K	−	Withdrawn	Yes	Yes	No	I
I32M/E48K	−	Withdrawn	Yes	Yes	No	I
G49A	−	Projecting	Yes	Yes	No	I
W51A	−	Projecting	Yes	Yes	No	I
T42A	+	WT	No	No	Yes	II
M52A	+	WT	Yes	Yes	Yes	II
S53A	+	WT	No	Yes	Yes	II
N72A	+	WT	No	No	Yes	II
L50A	−	WT	No	No	Yes	III
C54A	−	Projecting	Yes	Yes	Yes	III
C64A	−	Projecting	Yes	Yes	Yes	III
Claudin-7 mutation						
WT	−	Withdrawn	Yes	Yes	No	I
M32I/K48E	+	WT	No	No	Yes	–

a WT – wild-type position; Withdrawn – moved away from CD81; Projecting – displaced into region occupied by CD81 in WT.

### Identifying CD81 residues that interact with claudin-1

**Fig. 3:**
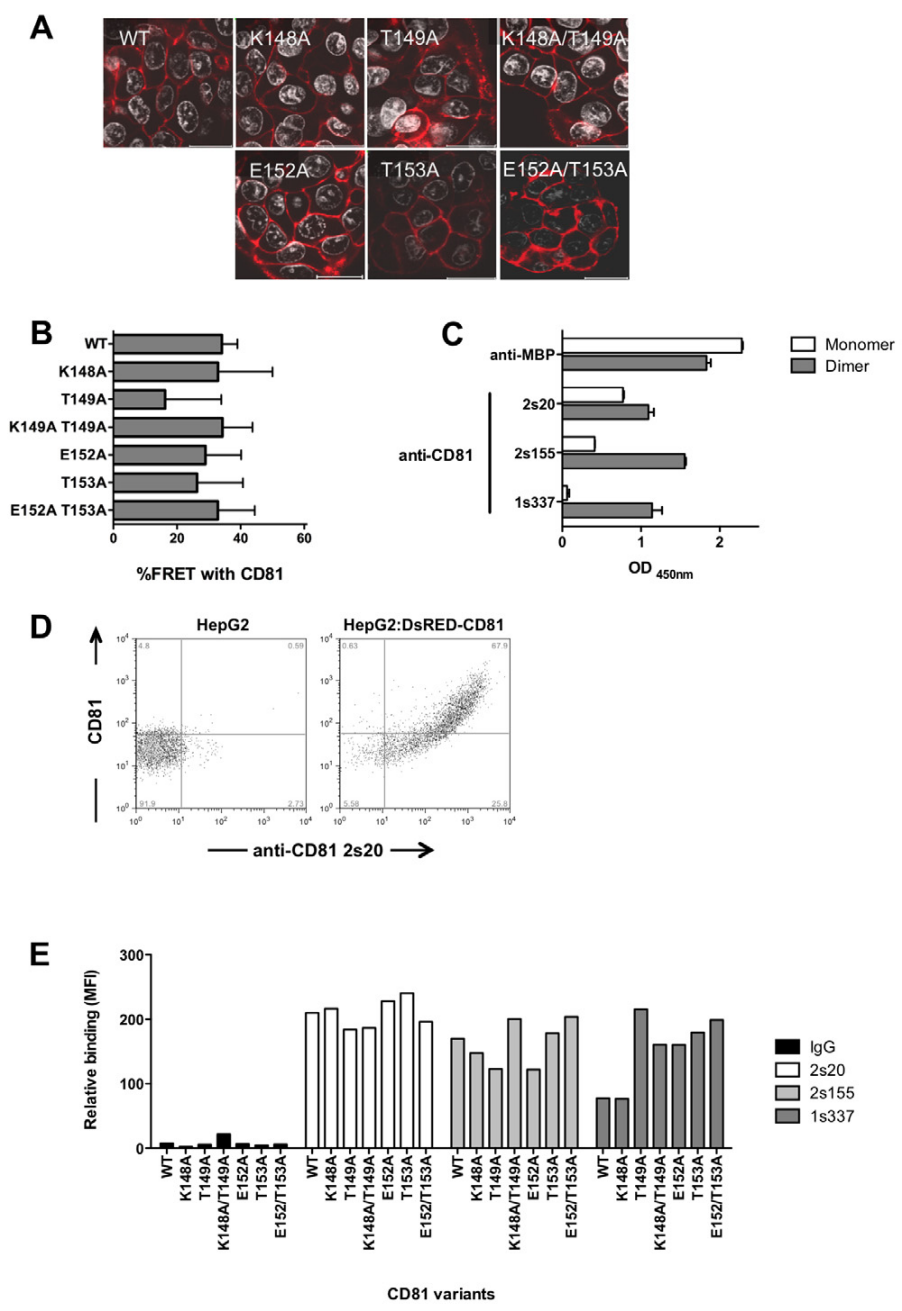
Effect of CD81 mutations on protein localization, antigenicity and HCV E2 binding. HepG2 cells were transduced to express wild-type (WT) DsRED-CD81 and mutant proteins and their localization assessed by confocal microscopy (A). %FRET between DsRED-CD81 mutants and AcGFP–CD81 in transfected 293-T cells, The mean values are derived from triplicate estimates which are representative of three further experiments (B). Anti-CD81 mAbs and rabbit anti-MBP were tested at 5 μg ml^−1^ for their reactivity with monomeric and dimeric MBP–CD81 by ELISA, where the data are presented as optical density at 450 nm (C). Flow cytometric binding of anti-CD81 2s20 to parental HepG2 and cells transduced to express DsRED-CD81 (D). Anti-CD81 mAb binding to HepG2 cells expressing WT DsRED-CD81 and mutant proteins. mAb binding is expressed as mean fluorescence intensity (MFI) relative to DsRED CD81 signals (E). The data presented are from a single experiment and are representative of two independent experiments.

Our model predicts that CD81 residues K148, T149, E152 and T153 interact with claudin-1 residues 63–66 (Fig. 1). To validate the model we substituted the selected CD81 residues to alanine and studied mutant protein association with claudin-1 and viral receptor activity. WT and mutant proteins were tagged at their N-terminus with DsRED and expressed in the CD81-negative HepG2 hepatoma cell line. All mutants localized to the plasma membrane, with minimal evidence of intracellular staining (Fig. 3A). Furthermore, we confirmed that none of the changes altered the ability of CD81 to dimerize with WT AcGFP–CD81 by FRET (Fig. 3B). We recently isolated a number of mAbs that recognize conformation-dependent epitopes in CD81 ECL2 that neutralize HCV infection (Farquhar *et al*., 2012). Among these mAbs we identified clones 2s155 and 1s337 that preferentially recognize dimeric CD81 ECL2 (Fig. 3C). In contrast, mAb 2s20 bound monomeric and dimeric CD81 with similar values (Fig. 3C). All three mAbs bound cell surface expressed CD81 (Fig. 3D), consistent with a dimeric CD81 structure (Seigneuret, 2006). We used these mAbs as tools to probe CD81 conformation by flow cytometry. Since all proteins were DsRED tagged anti-CD81 mAb binding was expressed relative to fluorophore expression (Fig. 3D). mAbs 2s155 and 1s337 bound HepG2 cells expressing WT and mutant CD81 proteins with comparable fluorescent intensities, demonstrating a native conformation for the mutants (Fig. 3E). Several CD81 mutants bound mAb 1s337 with greater fluorescent intensities compared with WT (Fig. 3E), suggesting a modest change in protein conformation.

**Fig. 4:**
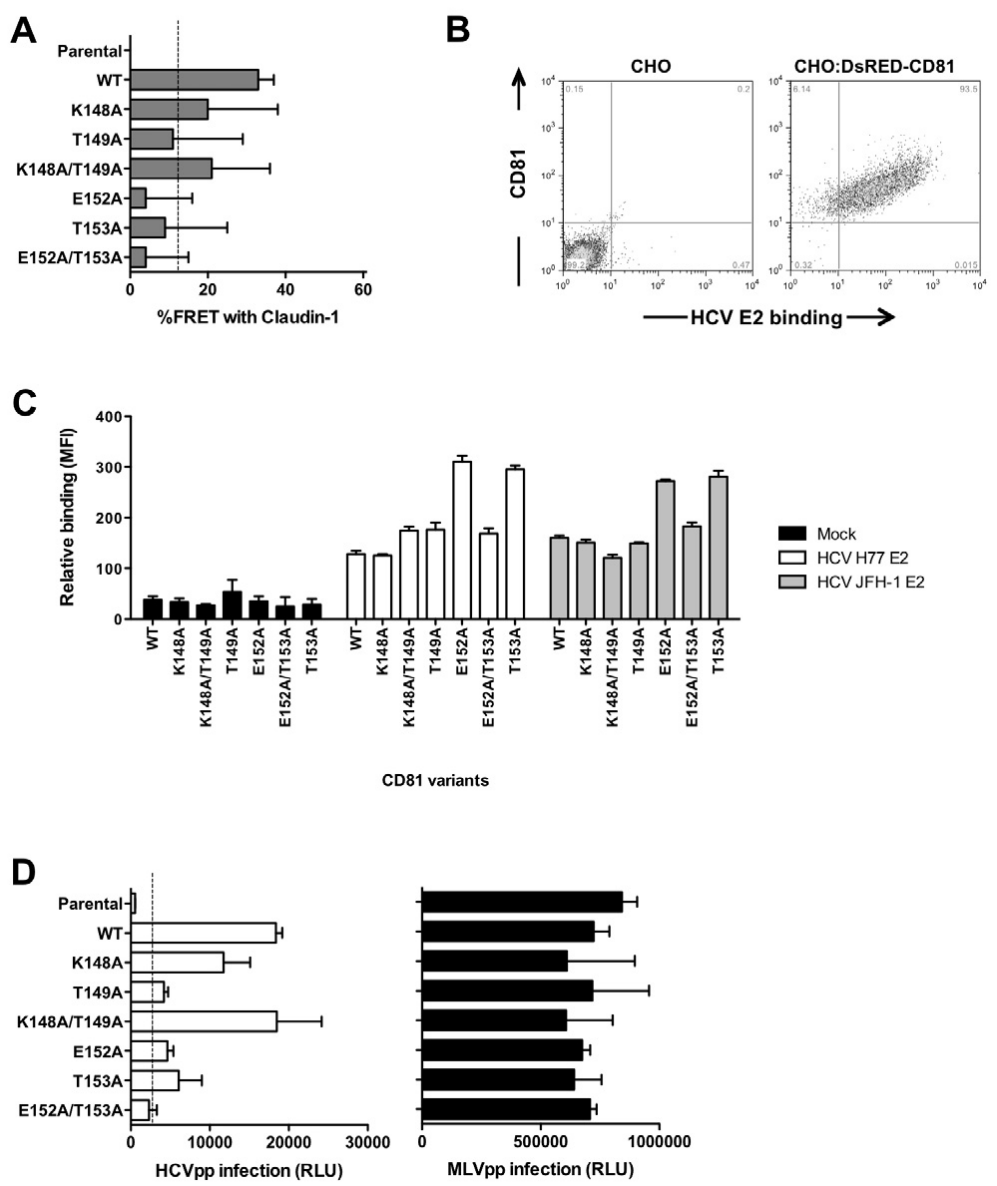
Effect of CD81 mutations on claudin-1 association and HCV infection. FRET between AcGFP–claudin-1 and DsRED-CD81 and mutant proteins expressed in 293-T cells (A). The mean plus 2SD of non-specific FRET values observed in parental non-transfected is indicated by the dashed line and represents the threshold for the assay. Flow cytometric binding of HCV E2 (strain H77) to parental CHO cells and those transduced to express DsRED-CD81 (B). HCV E2 strain H77 and JFH-1 binding to CHO cells expressing wild-type (WT) DsRED-CD81 and mutant proteins. HCV E2 binding is expressed as mean fluorescence intensity (MFI) relative to DsRED CD81 signals (C). HepG2 cells were transduced to express WT DsRED-CD81 and mutant proteins and evaluated for their ability to support HCVpp or MLVpp infection (D). Flow cytometry of transduced HepG2 cells confirmed comparable levels of CD81 expression. Data are expressed as specific infectivity where the value of an envelope deficient pseudoparticle is subtracted from both HCVpp and MLVpp relative light unit (RLU) signals. The dashed line represents the mean plus 2 SD of HCVpp infection of CD81-negative parental HepG2 cells; levels of infection below this threshold are considered negative. The data presented are from a single experiment and are representative of two independent experiments.

Mutation of T149, E152 and T153 residues to alanine reduced CD81 FRET with claudin-1, whereas replacement of residue K148 had no significant effect (Fig. 4A). These data confirm that the main loop interactions involve T149, E152 and T153, but that K148, although within reasonable range for interaction may play a role in the steric fitting of the two loops rather than in specific interactions with claudin-1 residues. To ascertain whether the mutations perturb CD81 interaction with HCV glycoprotein E2, CHO cells were transduced to express DsRED tagged mutants and evaluated for their ability to bind HCV strains H77 and JFH-1 E2 glycoprotein by flow cytometry. HCV E2 glycoproteins bound to CHO cells expressing WT and mutant CD81 (Fig. 4C), suggesting a minimal perturbation of the HCV E2 binding site. HEK 293-T cells naturally lack claudin-1 expression and transduction to express claudin-1 renders them sensitive to HCVpp entry (Evans *et al*., 2007). Comparable levels of HCV E2 bound to 293-T and 293-T-claudin-1 by flow cytometry (Mean FI of 62 and 79 respectively), suggesting that claudin-1 expression does not modulate CD81-E2 association. It is worth noting that CD81 mutants E152A and T153A bound higher levels of both E2 glycoproteins. HepG2 cells expressing T149A, E152A or T153A CD81 mutants showed minimal evidence for HCVpp infection, whereas cells expressing K148A or K148A/T149A CD81 supported virus infection at comparable levels to WT CD81 (Fig. 4D). MLVpp infected parental and mutant CD81 expressing HepG2 cells at comparable levels (Fig. 4D). In summary, these data confirm the essential role for T149, E152 and T153 residues of CD81 in virus entry and association with claudin-1.

## Discussion

We applied a bio-informatic modelling-based mutagenesis approach to characterize the CD81/claudin-1 extracellular loop interactions essential for HCV infection. Our *in silico* model identified interacting residues between CD81 and claudin-1 and *in vitro* mutagenesis confirmed their role in HCV entry. We predict that the first extracellular loop of claudin-1 is relatively unstructured, with a high beta turn composition and two motifs, incorporating amino acids 33–35 and 63–66, that interact with CD81. Previous reports have shown that mutation of claudin-1 ECL1 residues 32 and 48 ablated CD81 association and virus entry (Harris *et al*., 2010), suggesting a direct role in receptor complex formation. However, *in silico* modelling of these variants showed the formation of an α-helical region, leading to a marked change in loop orientation, providing an alternative explanation for their inability to associate with CD81 and failure to support HCV infection.

To validate our *in silico* model we studied a panel of claudin-1 mutants, previously reported to modulate HCV entry (Cukierman *et al*., 2009). We demonstrate a significant association between claudin-1 viral receptor activity and interaction with CD81 *in silico* and *in vitro*. Three claudin-1 mutants (L50A, C54A and C64A – group III) were predicted to interact with alternative CD81 residues L162, I182, N184 and F186. All three variants displayed significant association with CD81 *in vitro* by FRET analysis and yet failed to support HCV entry, demonstrating that this altered orientation of the two proteins is receptor inactive. Of note, all of these residues were previously reported to be involved in binding HCV E2, suggesting that the second extracellular loop of CD81 needs to interact with both HCV E2 and claudin-1 to confer virus entry (Drummer *et al*., 2002; 2005).

*In silico* modelling of claudin-7 ECL1 demonstrated minimal contact with CD81 (Fig. 1E), with the loop projecting outwards in a similar fashion to the group I claudin-1 mutants. This projection fills the space that would be occupied by the α-helix containing CD81 residues T149, E152 and T153. Introduction of M32I/K48E changes into claudin-7 (Fig. 1F) re-orientates the loop and allows claudin-7 C64 to interact with CD81 E152 in a similar way to that seen for WT claudin-1.

Mutagenesis studies have shown the importance of the highly conserved claudin motif, W30–GLW51–C54–C64 in HCV entry (Cukierman *et al*., 2009). Our data provide a structural rationale for these observations. Mutation of W30, G49 or W51 induced substantial changes in the local conformation and orientation of the ECL1 motifs predicted to interface with CD81, such that group I claudin-1 mutants disrupt the interaction of region 63–66 with CD81 (Table 1). In contrast, mutation of claudin-1 residues L50, C54 or C64 resulted in more modest changes to ECL1 orientation, leading to an interaction with alternative CD81 residues.

In forming tight junctions, claudins form homodimers between identical claudin molecules and heterodimers with different claudin family members. The intermolecular interface of claudin-5 has been shown to include both aromatic (F147, Y148, Y158) and hydrophilic (Q156, E159) residues in ECL2 (Piontek *et al*., 2008). Given the conserved nature of these residues across the claudin family, our model is consistent with distinct roles for the extracellular loops in dimerizing with other claudins and associating with tetraspanins (Kovalenko *et al*., 2007). Our model predicts that a claudin-1 protein is capable of simultaneous association with other claudin proteins and CD81.

We confirmed that mutation of CD81 residues T149, E152 and T153 to alanine reduced claudin-1 association and HCVpp entry. In contrast, mutation of residue K148A had no effect on CD81 interaction with claudin-1 *in vitro* and supported HCVpp infection. Importantly, a panel of conformation-dependent anti-CD81 antibodies that neutralize HCV infection (Farquhar *et al*., 2012) showed comparable binding to all mutants. These data, together with the observation that HCV E2 protein bound mutant and parental CD81 with comparable values, suggest that the mutations have minimal effect on CD81 conformation. However, a CD81 variant bearing the double mutation K148A/T149A was receptor active, suggesting that K148A suppresses the receptor inactive T149A mutant. Our model predicts that CD81 K148 interacts with claudin-1 Y33, whereas T149 interacts with claudin-1 motif 63–66. Importantly, the K148A/T149A double mutation reinstates the interaction of CD81 residue E152 with claudin-1 Q63. Although murine CD81 differs from human CD81 at 17 of the 87 residues of the ECL2 domain, the region identified here is conserved, leading us to speculate that the functions defined by this motif are similar in both organisms (Flint *et al*., 2006). These experimental results validate our structural predictions and highlight claudin-1 region 63–66 interaction with T149 and E152 of CD81 as the site of primary molecular association. In contrast, the 33–35 region of claudin-1 appears to determine the orientation and packing of the claudin-1 and CD81 extracellular loops.

Importantly, all of the CD81 mutants studied (K148, T149, E152 and T153) bound HCV E2, consistent with a report by Drummer and colleagues that residues outside this region form the HCV E2 binding site (Drummer *et al*., 2002). It is interesting to note that these residues, which were identified by *in silico* modelling and verified by site-directed mutagenesis, have previously been shown to have a role in the association between the ECL1 and ECL2 domains of CD81, suggesting that this region may have a role in both homotypic and heterotypic interactions of CD81 (Yalaoui *et al*., 2008). Our data suggest that distinct regions of CD81 ECL2 engage HCV glycoproteins and claudin-1, highlighting the potential to form a ternary HCV/CD81/claudin-1 complex. Although claudin-1 does not appear to modulate the ability of CD81 to bind HCV E2 (data not shown), claudin-1 limits CD81 lateral movement at the plasma membrane and promotes receptor endocytosis (Farquhar *et al*., 2012; Harris *et al*., 2012), suggesting a role for the CD81–claudin-1 complex in virus internalization and fusion with early endosomes. Our data uncover a novel role for CD81 residues T149, E152 and T153 in HCV entry independent of viral glycoprotein-receptor interaction, and substantiate the key role of CD81–claudin-1 complexes in HCV internalization. Our model of the CD81/claudin-1 interface provides a new conserved target for anti-viral drug design and will allow the rational design of small molecule and peptide mimetics targeting the receptor complex.

## Experimental procedures

### Structural modelling

The CD81/claudin-1 complex structure was modelled using a homology pipeline (http://membraneproteins.swan.ac.uk/modelling/), assembled in the Biskit structural bioinformatics platform (Grunberg *et al*., 2007) and scans the entire PDB database for candidate homologues. The pipeline incorporates the NCBI tools platform (Wheeler *et al*., 2008), the blast program (Altschul *et al*., 1990) for similarity searching of sequences, t-coffee (Notredame *et al*., 2000) for alignment of candidate sequences with the template, the modeller program (Eswar *et al*., 2003) for model assembly and the dssp algorithm (Kabsch and Sander, 1983) for secondary structure validation. This pipeline has been employed in the structural modelling of several membrane protein families (Sanner *et al*., 1996; Yang *et al*., 2009; Chung *et al*., 2010; Mullins *et al*., 2010; 2011). Homology models were generated using 10 iterations of the modeller program. Interactions between the structural models of claudin-1 WT or mutants and known CD81 crystal structure (PDB: 1G8Q) (Kitadokoro *et al*., 2001) were simulated using Hex 5.0 (Mustard and Ritchie, 2005), optimally fitting for shape and electrostatic interactions. Surface areas were calculated using the msms package (version 1.3; Sanner *et al*., 1996).

### Cell lines

293-T, HepG2 and CHO cell lines were maintained in Dulbecco's modified Eagle's medium (DMEM) supplemented with 10% fetal bovine serum, 1% l-Glutamine and 1% non-essential amino acids (Invitrogen, Carlsbad, CA).

### Genesis and fluorophore tagging of CD81 and claudin-1

Claudin-1 mutants were kindly provided by Tatjana Dragic (Albert Einstein University, NY) and open reading frames (ORFs) amplified with Phusion™ High-Fidelity DNA Polymerase (New England Biolabs) with gene-specific primers (Table S1). Amplicons were cloned into pJET2.1 plasmid (CloneJET system, Fermentas, UK), sequenced and subcloned into either pBABEpuro or pTRIP (Zennou *et al*., 2001; Harris *et al*., 2010). An AcGFP or DsRED fluorophore coding region was inserted at the N-terminus of the protein (Harris *et al*., 2010). CD81 mutants were generated using a series of mutagenic primers designed to exploit silent SalI or SacI sites in the CD81 ORF (Table S1). The 5′ and 3′ halves of CD81 were amplified, digested and ligated into pJET2.1 to produce full-length CD81 ORFs encoding the desired mutations that were transferred into pTRIP-AcGFP or pTRIP-DsRED plasmids and sequenced.

### Measurement of interaction between CD81 and claudin-1 by mammalian two-hybrid association

The WT CD81 and mutated claudin-1 molecules were transferred into pBIND-RLuc(GAL4) and pACT(VP16) vectors respectively (Promega ‘Checkmate’ system). Interaction between the two proteins was detected *in vitro* by co-transfecting 293-T cells with these constructs and a GAL4/VP16 driven pG5FLuc reporter plasmid, the levels of Renilla luciferase obtained from the pBIND vector acts as an internal control for transfection efficiency. Known interacting proteins (pBIND-Id and pACT-MyoD) and empty vectors were used as positive and negative controls respectively. After 24 h interaction between the constructs was detected by measuring levels of Firefly luciferase (FLuc), in accordance with the manufacturer's instructions (Promega).

### Pseudoparticle generation and infection

Pseudoviruses expressing a luciferase reporter were generated as previously described (Hsu *et al*., 2003). Briefly, 293-T cells were transfected with a 1:1 ratio of plasmids encoding HIV provirus expressing luciferase and HCV strain H77 E1E2 envelope glycoproteins (HCVpp) and Murine Leukaemia virus envelope glycoprotein (MLVpp) or empty vector (Env-pp). Supernatants were harvested 48 h post transfection, filtered and virion associated p24 determined using a commercial assay (Aalta Bioreagents). Infection was quantified by measuring luciferase activity (relative lights units, RLU) and specific infectivity determined by subtracting the mean Env-pp signal from the HCVpp and MLVpp values.

### CD81 and claudin-1 FRET analysis

FRET analyses were performed as previously described (Harris *et al*., 2010). Briefly, 293-T or HepG2 cells were transfected to express the fluorescent-tagged CD81 and claudin-1 proteins, grown on 22 mm diameter borosilicate glass coverslips and imaged using a Zeiss MetaHead LSM confocal system. FRET was assessed before and after photobleaching at 561 nm to ablate AcGFP to DsRED energy transfer. Using the microscope's built in profiling function, the fluorescence intensities of several hundred pixels at the plasma membrane were determined at the light frequencies of the two fluorophores and the stoichiometry of labelled proteins measured as previously described (Zheng and Zagotta, 2004; Staruschenko *et al*., 2005; Harris *et al*., 2010). When regression analysis indicated a significant association between the fluorophores we assessed the occurrence of FRET between the pixels using a gradual acceptor photobleaching method (Zal and Gascoigne, 2004). Briefly, the fluorescence intensity of the AcGFP fluorophore (the donor fluorophore) was determined before and after photobleaching, with an increase in signal indicating the occurrence of FRET. The frequency of pixels in which FRET was detected gives a measure of AcGFP–DsRED interaction, recorded as the percentage FRET (Harris *et al*., 2008). Statistical analyses were performed using a non-parametric one-way anova (Kruskal Wallis test) or Student's *t*-test in Prism 4.0, where necessary corrections for multiple comparisons were made.

### Anti-CD81 mAbs

Anti-CD81 mAbs 1s337, 2s20 and 2s155 were generated following immunization of mice with recombinant full-length CD81 (Jamshad *et al*., 2008). Purified mouse immunoglobulin was a gift from M. Goodall (University of Birmingham). CD81 MBP-LEL113-201 (MBP–CD81) was expressed in *Escherichia coli* strain BL21 cells as previously reported (Drummer *et al*., 2002). Bacteria were lysed by sonication in S-buffer (300 mM NaCI, 100 mM Tris pH 8.0, 1 mM EDTA, 0.02% sodium azide) and cell debris pelleted by centrifugation at 39 000 *g* for 30 min. MBP–CD81 fusion proteins were affinity-purified from the soluble fraction using amylose-agarose (New England Biolabs) as recommended by the manufacturer and proteins eluted with 10 mM maltose. Monomeric and dimeric MBP–CD81 were isolated by Superdex 200 gel filtration chromatography (Amersham) and protein purity assessed by SDS/PAGE. mAbs were screened for their reactivity with native and heat denatured MBP–CD81 and 15-mer peptides overlapping CD81 ECL2. All mAbs failed to interact with peptides or denatured protein, leading us to conclude that they recognize conformation-dependent ECL2-specific epitopes.

### CD81–HCV E2 binding

To determine the effect of CD81 mutations on HCV E2 glycoprotein binding, WT or mutant CD81 were expressed in CHO cells using the pTRIP expression system. CHO cells do not bind HCV E2 and provide a model system to study exogenous human receptor affinity for HCV E2. Cells were seeded at 2 × 10^5^ cells per well in a 96 U bottomed well plate in PBS/0.5% BSA/0.01% sodium azide for 20 min, incubated with HCV strain H77 and JFH-1 E2_661_ tagged at the C-terminus with a HIV epitope tag at 5 μg ml^−1^ diluted in PBS/0.5% BSA/0.01% sodium azide and incubated for 45 min at 37°C. Unbound HCV E2 was removed by washing with PBS and bound antigen detected with mAb 10/76b, which recognizes a HIV gp120 epitope tag (Flint *et al*., 1999). After 45 min at 37°C the cells were washed and incubated with a 1:1000 dilution of an Alexa-633 ant-Rat IgG (Invitrogen, Carlsbad, CA) for a further 45 min at 37°C. Cells were washed, fixed with 4% paraformaldehyde and bound HCV E2 visualized by flow cytometry.

## Supporting information

**Table S1.** Primers used in genesis of claudin-1 and CD81 mutants.
