# Supplementary material for: In silico directed mutagenesis identifies the CD81/claudin-1 hepatitis C virus receptor interface
Source: Cell Microbiol. 2012 Sep 25;14(12):1892–903. doi: 10.1111/cmi.12008 (PMC3549482; doi:10.1111/cmi.12008)
Supplement: Supplementary file 1 [file cmi0014-1892-SD1.pdf]

Supplementary Table 1 : Primers used in genesis of Claudin-1 and CD81 mutants

Primer Name[1]	Primer Sequence[2]
**Claudin-1 primers**	
Claudin-1 FWD (+)	ggatccGCCACCATGGCCAACGCGGGGCTG
Claudin-1 REV (–)	ctcgagTCACACGTAGTCTTTCCCGCTGG
**CD81 Primers**	
CD81 FWD (+)	TCTAGAggatccGCCACCATGGGAGTGGAGGGCTGCAC
CD81 K148A (–)	CAGTgtcgacGAGCCACAGCAGTCAAGCGTCTCGTGGAAGGTCgcCACCAC
CD81 T149A (–)	CAGTgtcgacGAGCCACAGCAGTCAAGCGTCTCGTGGAAGGcCTTCACCAC
CD81 K148A/T149A (–)	CAGTgtcgacGAGCCACAGCAGTCAAGCGTCTCGTGGAAGGcCgcCACCAC
CD81 E152A (–)	CAGTgtcgacGAGCCACAGCAGTCAAGCGTCgCGTGGAA
CD81 T153A (–)	CAGTgtcgacGAGCCACAGCAGTCAAGCGcCTCGTGGAA
CD81 E152A/T153A (–)	CAGTgtcgacGAGCCACAGCAGTCAAGCGcCgCGTGGAA
CD81 K201A (–)	TGCCGATGAGGTACAgagctcCGGAGAAGAGG
CD81 155-166 (+)	ggatccTGCTGTGGCTCgtcgacACTGACTGCTTTGAC
CD81 184-207 (+)	ggatccCCTCTTCTCCGgagctcTGTACCTCATCGGCAT
CD81 REV (–)	TTTCTAGGTctcgagTCAGTACACGGAGCTGTTCCGG

1) FWD or (+) sense primer, REV or (–) antisense primer
2) Restriction enzyme sites shown in lower case, Start and Stop codons underlined.
