# Supplementary figures and images for: In silico directed mutagenesis identifies the CD81/claudin-1 hepatitis C virus receptor interface
Source: Cell Microbiol. 2012 Sep 25;14(12):1892–903. doi: 10.1111/cmi.12008 (PMC3549482; doi:10.1111/cmi.12008)

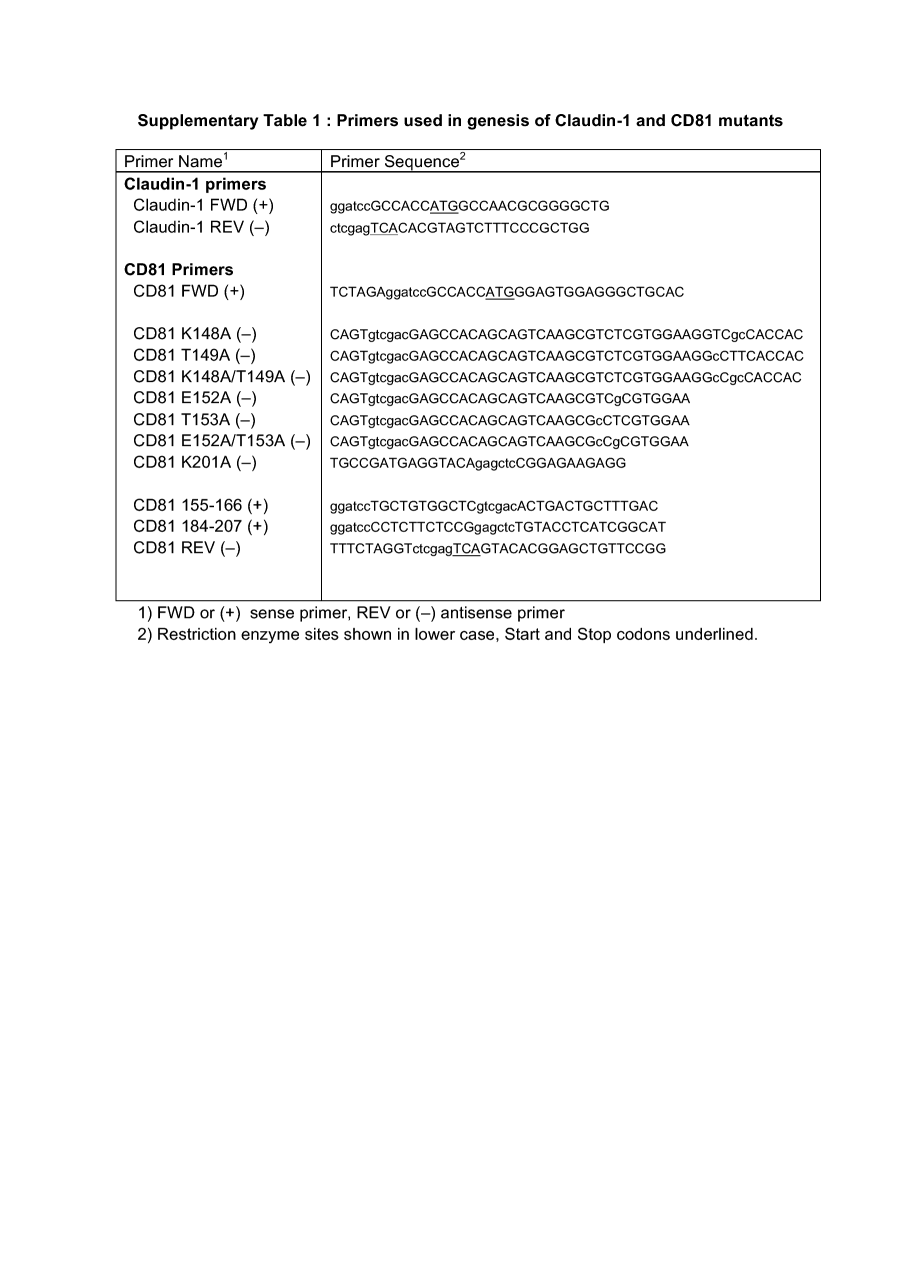

Supplement: Supplementary file 2 [file cmi0014-1892-SD2.png]
